# A coach leadership reflection tool: facilitating coach reflection as a pathway for interpersonal development

**DOI:** 10.3389/fpsyg.2026.1894977

**Published:** 2026-09-16

**Authors:** Mia KurtzFavero, Stuart G. Wilson, Jean Côté

**Affiliations:** Performance Lab for the Advancement of Youth in Sport, School of Kinesiology and Health Studies, Queen’s University, Kingston, ON, Canada

**Keywords:** coach education, coach observation, coach-athlete relationships, global rating scale, intrapersonal knowledge, professional development, reflective practice, youth sport coaching

## Abstract

This study draws on models from medicine and education to examine competitive youth coaches’ perceptions of a reflective intervention on their interpersonal behaviours, which included pre-briefing, in-situ observation, reflection cards, and debriefing sessions. Through qualitative interviews with 12 coaches conducted before and after the intervention, we explored its usability and the coaches’ perceived outcomes in awareness, confidence, motivation, and perceived behaviour change related to interpersonal and intrapersonal coaching practices. Findings highlight the transformative potential of a reflective intervention to support meaningful behaviour change and enhance coaching effectiveness. Coaches reported increased confidence and a deeper appreciation of the role of interpersonal and intrapersonal skills in fostering positive coach–athlete relationships following the intervention. These insights offer important implications for coach development programming and applied coaching practice.

## Introduction

Coaches play a crucial role in shaping the experiences of youth athletes, particularly through the influence of positive coach-athlete relationships (i.e., interpersonal interactions) on psychosocial benefits such as improved social skills, team cohesion, and intrinsic motivation (Adie and Jowett, 2010; Evans et al., 2015; Fraser-Thomas et al., 2005). Relatedly, coaches can strengthen their coach-athlete relationships by engaging in structured reflection to help critically evaluate their behaviours, refine decision-making, and increase responsiveness (Nelson and Cushion, 2006; Whitehead et al., 2016). Such skills may be developed through coach education programs, whereby studies have shown increased coaching effectiveness and improvements in athlete outcomes (e.g., positive self-perception, intrinsic motivation, long-term sport participation) as a result of coach education program interventions (Li et al., 2025). Extant research also suggest that coach education programs designed to enhance coaches’ interpersonal effectiveness can positively impact athletes’ cognitive outcomes (e.g., reduced anxiety, increased mastery orientation; Langan et al., 2013). Despite these benefits, the existing literature indicates that many formal coach development programs offered in Canada, United Kingdom, United States, and Australia largely focus on training sport-specific skills (92%; Lefebvre et al., 2016), while comparatively fewer emphasize the types of reflective (2%) and interpersonal skills (6%) that may improve coaching effectiveness by enhancing coaches’ relational competencies (Côté and Gilbert, 2009; Lefebvre et al., 2016). A recent review of formal coach education programs delivered by national governing bodies (Wang et al., 2025), highlighted coaches’ criticisms of coach education content, noting that it often emphasizes the *what* of coaching (technical and tactical knowledge) at the expense of the *how* (interpersonal and intrapersonal knowledge).

Coaching effectiveness can be defined as the consistent application of coaching knowledge to improve athlete outcomes in relevant coaching contexts (Côté and Gilbert, 2009). Specifically, the definition includes three components: (1) coaches’ knowledge and behaviours (i.e., professional, interpersonal, and intrapersonal), (2) athletes’ outcomes (competence, confidence, connection, and character), and (3) coaching contexts (recreational, developmental, and elite sport). From a coach education perspective, their definition highlights three knowledge areas that, when effectively applied, each play a vital role in building a comprehensive coaching skill set. Côté and Gilbert (2009) situate professional knowledge and behaviours as sport-specific understanding and technical/tactical instruction. Interpersonal knowledge and behaviours are explained as a coach’s ability to develop and maintain personal relationships, communicate effectively, and manage coach-athlete interactions. Lastly, intrapersonal knowledge and behaviours refer to a coach’s capacity for critical reflection on personal behaviours and practices. Intrapersonal knowledge and behaviours are essential for applying self-assessment to identify strengths, weaknesses, and to enhance future coaching approaches (Whitehead et al., 2016). Accordingly, strong coach-athlete relationships are built upon the effective application of interpersonal knowledge, which may be developed through the effective application of intrapersonal knowledge and behaviours (Côté and Gilbert, 2009). Overall, coaches’ interpersonal and intrapersonal knowledge and behaviours are fundamental to fostering strong coach-athlete relationships (Lefebvre et al., 2016; Zhao and Jowett, 2023).

Consequently, various studies in coach education have examined how reflective practices impact coach learning and development (Cropley et al., 2015; Nelson and Cushion, 2006; Whitehead et al., 2016). Overall, participating in reflective processes boosts coaches’ understanding of their knowledge, behaviours, and coaching philosophies and encourages the adoption of innovative teaching and learning methods. Furthermore, these processes motivate coach educators and mentors to promote reflective practices and evaluations with their coaches. Nelson and Cushion (2006) emphasize the importance of integrating reflective practice into coach education and development programs to link experience with knowledge. Positioning that regular reflective practice is vital for improving individuals’ awareness of their coaching context. Accordingly, previous sport coaching literature has explored how coaches engage in reflective practices (Raya-Castellano et al., 2021; Rodrigue and Trudel, 2018). For example, approaches to coach reflection include journals, mind maps, reflective conversations, visual and audio prompts (Cropley et al., 2015), and reflective cards (Gilbert, 2017; Rodrigue and Trudel, 2018). Although such reflective practices are implemented through coach education and development programs, it is important to note that much of this work currently focuses on technical and tactical pedagogical skills (Raya-Castellano et al., 2021; Rodrigue and Trudel, 2018), which overlooks a key component of effective coaching: interpersonal knowledge, and relationships.

More generally, reflection is a mental process in which individuals become aware of and examine their personal beliefs, experiences, and behaviours (Schön, 1983). Reflective practice in professional and educational settings enables individuals to develop critical thinking and problem-solving skills while adapting to unique situations. Opportunities for reflection in coaching are wide-ranging and can involve scenarios such as role conflicts, performance tactics, team dynamics, game strategies, and developmental consequences (Gilbert and Trudel, 2001). Previous research identifies three types of reflection: reflection-in-action (during activity), reflection-on-action (between activities), and retrospective reflection-on-action (after completion of activity; Gilbert and Trudel, 2001; Schön, 1983). Additionally, most studies conducted with coaches assess coaches’ reflections on their professional behaviours (Cropley et al., 2015; Gilbert, 2017; Rodrigue and Trudel, 2018).

Meanwhile, interventions with physicians and teachers have used specific reflection tools that focus on the interpersonal dynamics they create in their teaching environments. Among potential frameworks for structured reflection, the *pre-brief*–*evaluation*-*debrief* model has been widely used in fields such as medicine (e.g., pre-surgery, surgical evaluation, post-surgery; Zhou et al., 2021) and education (Ministry of Education, 2022) to enhance performance and learning. In the surgical environment, where there is increased risk, teaching and evaluation methods are well studied, with much of the literature emphasizing a pre-brief, intraoperative teaching, and debrief model to develop teaching and leadership competencies (Pan et al., 2023; Zhou et al., 2021). A comparable structure exists in the current Ontario (Canada) education system, where teacher performance reviews follow a structured approach (i.e., pre-observation meeting, observation, post-observation meeting; Ministry of Education, 2022). These stages clarify goals and expectations and facilitate feedback on competencies, strategies, and areas for growth. Together, medical and education practices offer transferable models of structured evaluation that coaching research can adapt to support coach reflection and the development of more effective interpersonal behaviours.

As discussed in research by Jowett (2017), Jowett and Ntoumanis (2004), Jowett and Poczwardowski (2007), and Zhao and Jowett (2023), high-quality coach-athlete relationships are critical, as they have been linked to various athlete outcomes (e.g., improved skill, personal growth, psychological well-being). As a result, conceptual frameworks, such as the Full-Range Leadership Model (FRLM; Bass and Riggio, 2006) have been established to better understand the impact of specific leadership behaviours. Since the FRLM effectively outlines a spectrum of leadership behaviours, observational instruments (i.e., the Coach Leadership Assessment System; Turnnidge and Côté, 2019) are needed to assess these behaviours within the sport context. Continuing the work on systematic coach observations (Turnnidge and Côté, 2019), Lefebvre et al. (2023) designed the Coach Leadership Assessment System – Global Rating Scale (i.e., CLAS-GRS) to assess coach leadership behaviours more globally. The CLAS-GRS aims to deliver an accessible evaluation of coach-athlete interactions that can be employed in various coaching contexts by a more comprehensive list of assessors (e.g., technical director, club administration, coach mentor, coach colleague) rather than solely trained researchers. The CLAS-GRS is considered more convenient and practical for real-world use in sport organizations, particularly for assessor-coach discussions and coach self-reflection. The tool was designed for in-situ use and applies a 7-point Likert potency scale to each leadership behaviour, accounting for the frequency, duration, and meaningfulness of coach-athlete interpersonal interactions. Assessment can be completed at a single event (e.g., practice) throughout three 10-min segments and encourages context description by including a section for observer feedback/notes.

The present study’s design was grounded in Lefebvre and colleagues’ work through adapting their CLAS-GRS coding template and following their established procedural guidelines (as previously described). Building on this foundation, the present research sought to address two gaps identified by Lefebvre et al. (2023) in the coach observation literature: (1) assess the CLAS-GRS’s ability to facilitate reflective discussions focusing on coaches’ interpersonal behaviours and (2) qualitatively examine the usability of a reflective intervention through collaboration with sport partners (i.e., coaches).

## Methods

This study implemented a four-phase reflective intervention (i.e., Coach Leadership Reflection; CLR), in which coaches participated in a pre-brief, in-situ observation, post-training reflection activity, and a debrief. Semi-structured interviews were used to capture coaches’ experiences with the intervention and were analyzed thematically.

### Philosophical underpinnings

This project utilized a pragmatic approach to address the study’s research questions. Pragmatism is a philosophical stance that emphasizes the practical consequences and utility of applied research questions, theories, methods, and findings (Giacobbi et al., 2005). Pragmatists prioritize the practicality of research and focus on co-producing knowledge, translating findings, and applying findings in real-world contexts (Morgan, 2007). Accordingly, the emphasis on practical consequences is reflected in the theoretical framework (i.e., reflection-on-action; Schön, 1983) used to conceptualize coach reflection as an active process to inform future action. The importance of intersubjectivity lies in the fact that both objective truths and subjective interpretations contribute value to research. By adopting a pragmatic stance, researchers prioritize the implications of the research questions and the completed research over adherence to the philosophical assumptions of specific methods (Giacobbi et al., 2005).

### Participants

Upon receiving ethics approval from the institute’s General Research Ethics Board, coaches were recruited through youth volleyball organizations in Ontario, Canada. Twelve competitive youth volleyball coaches representing a range of athlete ages/genders, years of coaching experience, and certification levels provided consent to participate in the present study (Table 1). All participants were actively coaching during data collection, and teams were in-season (i.e., conducting at least two practices per week and travelling for games/tournaments). Additionally, coach participants were their team’s ‘head/lead coach’ (hereby referred to as coach). For the present study, ‘head coach’ was defined as the coach who holds the greatest responsibility on the coaching staff and is designated as the head coach on the team’s official roster. These inclusion criteria ensured that participants had authority and autonomy over the flow of their training sessions (i.e., practices), and their interactions with their athletes.

**Table 1:** Participant demographics.

Coach	Gender	Athlete gender	Athlete age	Coaching experience	Years with current team	Certification
C1	Male	Male	Under 13	1–5 years	<1 year	Level 1
C2	Male	Female	Under 14	1–5 years	<1 year	Level 1
C3	Female	Female	Under 14	1–5 years	<1 year	Level 1
C4	Female	Female	Under 14	6–10 years	2–4 years	Level 1
C5	Female	Female	Under 15	6–10 years	2–4 years	Level 2
C6	Female	Male	Under 16	11 + years	5 + years	Level 1
C7	Female	Female	Under 16	6–10 years	2–4 years	Level 1
C8	Female	Female	Under 16	6–10 years	<1 year	Level 1
C9	Male	Male	Under 16	11 + years	<1 year	Level 1
C10	Male	Female	Under 18	11 + years	5 + years	Level 2
C11	Male	Male	Under 18	11 + years	<1 year	Level 2
C12	Male	Female	Under 18	11 + years	<1 year	Level 3

### Procedure

#### Reflective intervention

The reflective intervention included four phases designed to stimulate reflection of interpersonal behaviours (reflection questions and reflection card available upon request). Each phase of the CLR is further detailed in KurtzFavero et al. (2026). To briefly summarize, phase one of the CLR—the *pre-brief*—involved familiarizing coaches with a range of interpersonal and leadership behaviours (i.e., FRLM behaviours), and situating the purpose of the intervention. Notably, the Ontario College of Teachers performance appraisals (Ministry of Education, 2022) and the National Coaching Certification Program (Coaching Association of Canada, 2016) include a pre-brief session in their respective processes. Following formats established by the Ontario College of Teachers performance appraisals (Ministry of Education, 2022) and the National Coaching Certification Program (Coaching Association of Canada, 2016), the CLR pre-brief involved discussing the tool’s expectations by gaining an understanding of the coaching context (e.g., “based on the leadership behaviours provided, which aspect is a strength for you?”). Phase two, the *in-situ observation,* employed the CLAS-GRS (available upon request) to systematically observe coaches’ leadership behaviours. The in-situ observations were conducted by the primary researcher, who was trained to employ the CLAS-GRS by a gold standard coder using previously coded footage and practiced coding in pilot observations prior to data collection. Phase three included the *post-training reflection card*, adapted from Gilbert’s (2017) version, where coaches independently completed an online self-reflection activity to prompt reflection on behaviours displayed during the observed practice, within the context of the CLAS-GRS items (e.g., “during training, did I demonstrate prosocial values?”). The fourth and final phase of the CLR was the *debrief*, which included a reflective discussion of the observed practice and the CLAS-GRS observations. Similar to the pre-brief, the debriefing questions were formulated to reflect the objectives (i.e., competencies, goals, strategies, areas for improvement) of the Ontario College of Teachers performance appraisal, post-observation meetings (Ministry of Education, 2022), and the NCCP’s (Coaching Association of Canada, 2016) certification debriefs (e.g., “which leadership behaviour do you think was an area of improvement for you in the observed session?”).

#### Data collection

The present study utilized semi-structured interviews and an open-ended questionnaire to collect data. The two semi-structured interviews were conducted immediately following the pre-brief (see Table 2) and debrief (see Table 3) sessions to encourage participants to provide feedback on their engagement with the intervention and insights into interpersonal/intrapersonal development as coaches. Semi-structured interviews were employed because of their ability to elicit rich, in-depth data (McIntosh and Morse, 2015), leverage participants’ capacity to expand on their responses, and elaborate through follow-up questions (Alsaawi, 2014). Furthermore, conducting interviews for intervention evaluation provides an opportunity to gain insight into the perspectives of those involved in the intervention, thereby allowing researchers to explore aspects that may not be observable (Patton, 1990).

**Table 2:** Pre-brief questions.

Section	Question
Reflection questions	How would you describe a typical interaction between yourself and an athlete? Based on the leadership behaviour items, what aspect of leadership behaviour is your strength? How do you foster this aspect of leadership behaviour during practices/games? Based on the leadership behaviour items, what aspect of leadership behaviour is an area of improvement for you? What is something you can do during your practice to work on your area of improvement? Do you regularly engage in any forms of self-reflection as a coach? What do you do? What is a leadership behaviour goal you have for your practice (tomorrow, etc.)?

**Table 3:** Debrief questions.

Section	Question
General CLAS-GRS results	What was a highlight for you during your practice? What was the most challenging part of the practice for you? Which leadership behaviour do you think was your greatest strength in the practice? Why? How did you engage in this behaviour? Which leadership behaviour do you think was an area of improvement for you? Why? How could you improve regarding this behaviour? Do you feel you met the goal you set in our pre-brief conversation of focusing on____________? What did you do to meet that goal? What would be one thing you could do during your next practice that would move you closer to meeting your leadership behaviour goal/continue working on an area of improvement? What would be the most difficult aspect to implement next practice that would move you closer to meeting that leadership behaviour goal/continue working on an area of improvement? Based on the observation assessment, is anything surprising to you? ___________was your most potent transformational leadership behaviour during your practice, what are your thoughts on that? (Do you feel that is typical for you? Why do you think it is the most potent behaviour?) ________ was your least potent transformational leadership behaviour during your practice, what are your thoughts on that? (Do you feel that is typical for you? Why do you think it is the least potent behaviour?) ________ was your most potent of the other leadership behaviours (i.e., toxic, transactional, laissez-faire), what are your thoughts on that? (Do you feel that is typical for you? Why do you think it was so potent for you?) Having seen the observation assessment, is there a goal you would like to set for future sessions? What is a behaviour you can do to help you achieve this goal? Are there any other reflections you had from the session that you would like to discuss?

All practice sessions lasted two hours each and were observed in person. All data collection sessions and interviews were conducted virtually using a video conferencing platform (i.e., Zoom™). Pre-brief sessions/interviews ranged from 22 min to 39 min in length, and debrief sessions/interviews ranged from 42 to 63 min. Qualtrics Experience Management Software (2020) was utilized to collect post-training reflection card data. While participants could withdraw from the study at any time without consequence, all interviews, observations, and surveys were completed in full. Before starting the pre-brief session, the participants were asked to read and sign the study’s letter of information and consent form. All interviews were recorded via Zoom™ for transcription. Any identifying information was removed or replaced with a pseudonym to ensure the participants’ privacy. Completed transcripts were uploaded to a qualitative analysis software (NVivo 14; Lumivero, 2021) for analysis.

The interview guides and questionnaire items were designed to align with Patton’s (1990) recommendations for interview content in qualitative evaluations. Thus, the study’s interview guides for both interviews are separated into four distinct sections: (a) *background/demographic*, (b) *opinions/values*, (c) *feelings*, and (d) *experiences/behaviour*. The first section, *background/demographic*, includes questions regarding coaching experience and coach education (e.g., “How long have you been coaching this specific team?”). Identifying participants’ histories as youth sport coaches provided context for the subsequent interview sections. The second section of the interview guide, *opinions/values*, explored participants’ cognitions and perspectives regarding the CLR intervention. An example question in this section of the guide includes, “How would you improve the current pre-brief process?” Section three, *feelings*, aimed to understand participants’ emotional responses to specific situations they experienced while engaging with the CLR (e.g., “Can you tell me about the emotions you felt when your practice was being observed?” In the final section of the interviews, the questions on *experiences/behaviour* were intended to assess participants’ experiences engaging with the CLR and their intent to implement or modify their coaching practices (i.e., interpersonal/intrapersonal behaviours). An example question in this section includes, “What is a key takeaway from the CLR that you want to share with a coaching colleague?”).

### Data analysis

The data were analyzed using an inductive reflexive thematic analysis, in which themes were constructed to represent the significant concepts and ideas identified in the data (Braun et al., 2016; Braun and Clarke, 2019; Braun and Clarke, 2021). Through this approach, researchers actively contribute to the development of themes, helping shape the study’s interpretations. Utilizing Braun and Clarke’s (2006) six-stage guide for thematic analysis and reflexive thematic analysis manual (Braun and Clarke, 2021), themes in this section were developed, analyzed, and interpreted through the following process.

The primary researcher first familiarized themselves with the dataset by listening to and transcribing the audio recordings, reviewed transcripts, and survey responses while creating notes. This process was particularly important for the qualitative survey responses, as the data were not generated through direct interaction with participants (Braun and Clarke, 2021). Interview transcripts and Qualtrics survey data were uploaded into NVivo 14 (Lumivero, 2021) for analysis. The primary researcher developed 18 initial codes capturing feedback on the CLR and experiences related to interpersonal and intrapersonal development. Through an iterative process, involving reviewing and discussing the initial codes, all members of the research team refined 12 candidate themes representing shared patterns of meaning across coaches’ experiences. A hand-written thematic map was then created to assess connections among themes, resulting in the amalgamation, separation, and removal of themes and the collaborative development of six final themes that coherently represented the dataset.

### Quality and rigour

To evaluate and enhance the methodological quality of this study, the research team adhered to Tracy’s (2010) eight criteria (worthy topic, rich rigour, sincerity, credibility, resonance, significant contribution, ethics, and meaningful coherence) for quality qualitative research. This study addressed a topic that is relevant, timely, and significant given that it directly responded to the call from Lefebvre et al. (2023) for further research on implementing the CLAS-GRS to stimulate reflective discussions. To enhance rigour, the study design was grounded in well-established theoretical frameworks, such as the FRLM and CLAS-GRS, as well as the four-phase intervention model adapted from medicine and education literature. Sincerity was demonstrated through the honesty and transparency provided by critical friends, who challenged assumptions and supplied an alternative lens when discussing and analyzing the data. To support credibility and minimize biases, the primary researcher engaged in reflexive journaling throughout the data collection and analysis process. The use of evocative participant quotations throughout the results demonstrates resonance and naturalistic generalizability, whereby readers – particularly coaches may relate to the complex experiences described by participants. In evaluating the significance of contribution of this research, we argue that the study extends the existing literature both theoretically and practically by responding to a gap in the coach observation literature and by offering an applied tool that can be adopted by sport organizations. This study addressed procedural (i.e., General Research Ethics Board approval), situational (i.e., consideration of methods and data sharing), and relational (i.e., reflection on researcher’s dual-role as facilitator and interviewer) ethical obigations. Finally, this study supports meaningful coherence by achieving what it set out to do; explore coaches’ perceptions of the CLR and its perceived impact on interpersonal and intrapersonal development.

## Results

Answering the study’s research question involved exploring participants’ perceived interpersonal and intrapersonal outcomes from engaging with the CLR intervention. The analysis process resulted in six themes representing coaches’ reflections on roles, challenges, professional development, awareness, confidence, and perceived behaviour change. Understanding coaches’ perceived outcomes provided qualitative insights to support an evaluation of the CLR intervention.

### “A moral responsibility”: the influential role of coach reflection

When considering the role that self-reflection plays within the complexities of being a sport coach, participants discussed the importance of regularly engaging in reflection as a means of continual growth and improvement: “I took this opportunity to think about maybe it is time I wake up and readjust my style” (C2). Notably, coaches recognized that to observe the developmental benefits of reflection, they must commit to introspection by way of identifying problems and areas for improvement. This idea aligns with the notion that sport-specific knowledge may constrain possible solutions for specific issues, whereas reflection can elevate coaches’ problem-solving abilities. C10 exhibited this concept:

I think reflection is the most powerful tool as a coach. You can know as much as you want about the game, but there’s so many other things that make teams and games go well. And when it does not go well, why? What is it? And then try to find that solution.

Coaches also stressed that reflection extends beyond personal development and also shapes how they support and influence their athletes. As C11 expressed:

Coaches are role models and they will most definitely influence other people. If you have a coach that not only reflects but teaches the opportunity and advantages of reflecting, now you have so many other people doing it.

This highlights a broader sense of moral responsibility, where C11 added coaches must “do [their] job to the best of [their] ability”, viewing reflection as an essential part of that role.

In sum, coaches expressed that reflecting on their behaviours is a key avenue for becoming better coaches by identifying areas of strength and improvement. Additionally, it was noted that coaches can act as role models for their athletes by engaging in self-reflection and discussing its importance with them. Coaches’ belief that reflection is a moral responsibility supports the call for practical intrapersonal development within applied sport settings.

### Time constraints and ego threat: barriers to coach reflection

Although many participants conveyed the importance of reflection, coach participants also expressed that reflection is generally intimidating. There are numerous aspects of reflection that make it daunting. To begin, many coaches cited being overwhelmed with responsibilities as a barrier to reflection. Participants stressed that coaching is an additional responsibility on top of their full-time employment. Therefore, the time to reflect is limited and doing so can be mentally exhausting. Reflection seemed to be a low priority as it was seen as overwhelming in combination with other life – and coaching – duties:

I teach, so I have 8 h a day which I’m responsible for that pays my salary. Coaching on top of that you are running to your practice, you are planning sessions, you are doing your coaching stuff. By the time you get back from practice, you want to rest and relax and not think about it or reflect, and sometimes it’s just hard making that time (C12).

In addition to immense responsibilities, participants identified ego as an unsettling aspect of reflecting on their coaching. Many expressed that it can be difficult to consider the aspects of their coaching that require improvement or situations they could have handled differently. Coaches take pride in their abilities and may, therefore, view mistakes as failures. This concept is demonstrated by C9: “I’m like, I was so stupid. This was all my fault. Why did I do this? It’s hard for a coach to not be bothered by it. And it’s also hard to admit to your own mistakes”. Recognizing and acknowledging one’s mistakes or areas of improvement was a prominent point of discussion amongst coaches; the idea that reflecting can “hit too close to home” (C4). The potential for reflection to affect coaches’ egos further perpetuates the intimidating nature of reflective practices for youth sport coaches: “talking about your weaknesses is hard and admitting to your faults is hard… people don’t want to admit when they need help or reflect on something they could have done better” (C7). Participants’ perceptions of reflection as disconcerting reinforce the need for greater, more accessible exposure to supports for coaches’ intrapersonal development.

### Something is missing: a gap in professional development

Coach participants noted a lack of exposure to interpersonal behaviours within coach development opportunities and felt it was an underrepresented area of training. Consequently, coaches struggled with understanding the balance between developing meaningful interpersonal relationships with their athletes, specifically through individualized consideration, and maintaining the principles of safe sport: “I want all my athletes to understand that I’m speaking to them as humans and so I try and make as many interpersonal connections as possible. But I have to keep space because they’re kids.” (C11). This finding highlights that coaches struggle with—or lack guidance on—how to effectively implement certain leadership behaviours in practice. While Canadian coaches are required to complete annual professional development through their national sport organizations (NSOs), participants noted that these opportunities often emphasize technical and tactical skills, with limited focus on interpersonal development. As C1 explained, interventions like the CLR shift the focus toward “developing human beings”, making them a valuable addition to existing systems. Beyond NSO requirements, participants also identified youth sport clubs as key spaces for ongoing education, suggesting that such interventions could support individual interpersonal growth while fostering conversations around club values and philosophies. Importantly, coaches emphasized the need to embed interpersonal and intrapersonal training within coach certification processes, ensuring early exposure to leadership behaviours and reflective practices.

In addition to identifying coach professional development needs, participants also recognized and appreciated the value of engaging in a reflective discussion with an external person (i.e., an individual not connected to the team). This was expressed as helpful for coaches because the opportunity provided a different perspective on their leadership behaviours: “Having an outside perspective, the idea of having another set of eyes and a voice to share was very helpful” (C10). Thus, another potential gap in coach professional development was identified. The concept of an outside perspective was discussed by many participants and deemed valuable as it may shed light on how athletes perceive coaches’ leadership behaviours – a key aspect of reflection and development that is somewhat difficult to obtain: “I can write down what I thought happened, but what you [observer] saw happened could be totally different. And what you saw is closer to what the athletes perceive. And that’s what we care about” (C1).

I’ll say for me [the tool was meaningful] by having more of a discussion about it, as opposed to just internal, cause you have your own perceptions of what your behaviours are, but somebody else might give you different feedback. So having that third party provide that input is important (C2).

Although coaches perceived regular reflection on their interpersonal behaviours as a key to continual growth and success, they felt that these areas were not a focus of coaching professional development. Coaches expressed the need for interpersonal and intrapersonal professional development opportunities and saw the CLR as an avenue for such growth.

### Fostering awareness and empowerment: the impact of CLR engagement

A prominent theme discussed by many participants was that the CLR raised awareness of various aspects of their coaching and “opened [their] eyes to be a more well-rounded coach” (C8). For instance, the coaches had minimal prior awareness of the FRLM and of specific coaching examples that can be applied to each behaviour. Several coaches noted that the CLR raised awareness of the FRLM behaviours (e.g., inspirational motivation) and practical examples of each (e.g., apologizing for mistakes as part of idealized influence), particularly through the slide deck presented during the pre-brief session: “unless coaches are specifically taught these [leadership behaviours], it is not something they would ever know about” (C5). Connecting the FRLM behaviours to specific behavioural examples allowed coaches to better understand the leadership dimensions, “see the bigger picture” (C9), and link the behaviours to real-world coaching situations.

Interestingly, many participants shared that the pre-brief session informally encouraged a mental checklist, in which coaches reflected on the leadership behaviours they do or do not engage in while those behaviours were being described to them. Through this unintentional mental activity, coaches discussed feeling self-assured that they were displaying positive interpersonal behaviours and “are on the right track” (C8).

As we were going through it [the pre-brief session], I was mentally ticking boxes off Like yep, yep, okay nice I do that… I was thinking about okay, I do that here and I do that there (C10).

Coaches who had strong convictions about the type of coach they wanted to be also reported feeling reassured by the in-situ observation that they were displaying the behaviours they intended and that aligned with their coaching philosophies: “when I look back, I think about what kind of coach I want to be and I really identified with the idealized influence part” (C5). Participants were inspired to reflect on whether their actual behaviours aligned with their intended leadership style and reported feeling self-assured when they coincided: “It made me feel good that I didn’t fall into toxic.” (C4). In this way, the awareness fostered by the pre-brief translated into coaches’ increased confidence in their existing interpersonal behaviours and coaching philosophies.

Beyond affirming existing behaviours, the CLR also helped coaches identify areas for growth. Coaches identified the in-situ observation and debrief session as a key factor in their reflection and recognition of areas of their coaching that could be developed: “I think for me to develop and evolve as a coach, this [debrief discussion about observed practice] gives me things to work towards and maybe point out things that I haven’t been doing, that I should be” (C7). Coaches also expressed that their engagement with the intervention provided awareness of leadership behaviours that could be incorporated into their coaching repertoire to enhance their interpersonal interactions and become a more transformational coach: “just seeing what behaviours I’m missing or could potentially add was really helpful. I want to know what else I can do to get better and more transformational” (C4). Reflecting on who one wants to be as a coach appeared to be an important part of this process; consequently, inexperienced coaches, or those trying to establish their values, may benefit from “figuring out which leadership behaviours are important to them” (C11), building both awareness and confidence in interpersonal behaviours and reflective skills.

Finally, many coaches recognized a need to reflect more on interpersonal interactions rather than focusing primarily on technical/tactical components. Notably, this gap only emerged when participants were specifically prompted, often leading to profound realizations about their reflective practices: “It’s [reflection] probably more about the actual drills and skills. I analyze what drills work well. I think I maybe need to be better at it [reflecting on interpersonal interactions]” (C6). Although coaches recognized the gap in their reflective practice, they appreciated identifying it and were eager to improve. Further, through the “structured reflection and specific questions” (C10), coaches were exposed to reflection strategies they could carry into future practice, leaving them feeling more confident in their ability to reflect meaningfully going forward. Overall, coach participants felt their engagement with the CLR was meaningful, as it provided several opportunities to become more aware of their coaching and leadership behaviours, and in turn, more confident in applying them in practice.

### A catalyst for growth: the CLR translating reflection into action

Encouragingly, all participants reported that their coaching or reflective behaviours would be affected in some way by their involvement with the CLR. More specifically, coaches were inspired by their participation and, therefore, motivated to change some of their interpersonal and/or reflective practices: “sport in our country would truly benefit from implementing this [CLR tool] into sport organizations” (C11). By identifying areas of improvement and strategies for reflection, as previously discussed, coach participants felt empowered and driven to implement what they had learned into their coaching practices. For instance, coaches shared their desire to implement the post-training reflection card into their regular reflective practices, as it would allow them to see their growth over time:

I think I will be more mindful of the impact I have on athletes every time I step into a gym… that is something I would like to take forward and this process [reflection card] will help me with that (C6).

Further, engaging in more frequent reflection on leadership behaviours was a key point of discussion amongst participant responses. Coaches noted that their participation would likely prompt more regular reflection, specifically on their interpersonal interactions: “I didn’t really reflect on practice in terms of how I was coaching, it was more technical/tactical. But, this will allow me to reflect more on my behaviour” (C7).

Engagement with the CLR also supported coaches in refining specific leadership behaviours (i.e., TFL) as participants continue their coaching careers. Participants highlighted how the intervention prompted ongoing self-improvement by encouraging them to integrate points discussed in the pre-briefs and debriefs into their reflection, noting that “taking one or two of these comments will help me in my reflection” and ultimately “make me a better coach” (C10). The CLR also helped coaches set and evaluate coaching goals, as one participant explained that learning about the FRLM was “helpful for knowing how to improve as a coach”, leading them to “set better goals…do more reflection, ask myself questions… so that we can be better” (C6).

## Discussion

The purpose of this study was to capture coaches’ feedback on a reflective intervention (i.e., CLR) and gain a deeper understanding of coaches’ experiences with interpersonal and intrapersonal development. The results show that the development of the CLR offer both researchers and practitioners a unique, streamlined, systematic method for coach development, with an emphasis on improving interpersonal behaviours – highlighting the need for future work on coach reflection, specifically on interpersonal behaviours. The discussion will consider coaches’ experiences with reflection and their feedback on the CLR tool outcomes (i.e., interpersonal and intrapersonal development). The final portion of the discussion will focus on academic and practical implications.

### Navigating the reflective landscape

The findings presented in this study underscore the ethical responsibility coaches feel to engage in reflective practices. Originating from an intrinsic desire for continual growth, reflection is perceived as a fundamental component of a coach’s role. Although the value of coach reflection is widely known (Cropley et al., 2015; Nelson and Cushion, 2006; Whitehead et al., 2016), little work appears to examine whether coaches themselves recognize the importance of adopting a reflective practice. In fact, work from Nash et al. (2022) demonstrates how some coaches view reflection as an inefficient use of their time and prefer immediate action. In contrast, the present findings indicate that coaches perceive reflection as a moral obligation, integral to their own development and that of their athletes. Furthering this work, the current research also indicates that the moral responsibility coaches feel they have stems from their belief that they have a duty to be role models for their athletes by discussing and demonstrating the importance of reflection.

A key point of discussion regarding the CLR intervention and reflection in general was that coaches reported feeling overwhelmed at times. The notion of coaches associating numerous barriers with reflection and professional development aligns with previous work (Coaches Association of Ontario, 2023; Pill et al., 2023). Although the current study echoes prior reports, the findings further indicate that coach reflection, particularly on interpersonal behaviours, can feel burdensome due to the demands of the youth sport coaching context. The vast majority (76%) of youth sport coaches in Ontario are volunteers (Coaches Association of Ontario, 2023) and only 4% of all Ontario coaches (including post-secondary and national) report being paid a salary. Additionally, it is estimated that approximately 27% of Canadian adults are sport volunteers – many of whom are coaches – dedicating an average of 105 h each year (Canadian Fitness and Lifestyle Research Institute, 2022). To this point, youth sport coaches’ time and energy are being pulled in many directions (i.e., full-time employment, family responsibilities, practice planning, and extra coaching duties); therefore, making extra time for reflection in addition to their other obligations seems daunting.

In addition to being overwhelmed with responsibilities, coaches in this study also reported feeling intimidated by the reflection process. Further extending the current literature, these feelings of intimidation originate from the vulnerability inherent in critically evaluating one’s own coaching practice, which often challenges the ego. The current study also advances this idea by identifying that the daunting nature of self-reflection is exacerbated by gaps in professional development programs, which lack training in reflective practices and interpersonal behaviours (Lefebvre et al., 2016). Thus, coaches are left to navigate their own interpersonal and intrapersonal development, often without the necessary tools or support; ultimately leading to feelings of inadequacy and apprehension. Without experience with reflective practices through CDPs, coaches are unable to develop the belief in their ability to be successful with these tasks. This belief, otherwise known as self-efficacy, is a key determinant of motivation and behaviour (Bandura, 1982). Therefore, it does not come as a surprise that coaches feel intimidated by reflection, as they have not been provided with the opportunities or resources to develop these skills. Consequently, this project’s findings support the call for an expanded delivery of interpersonal and intrapersonal coach development programming (Da Silva et al., 2020; Lefebvre et al., 2016).

### Tools for life

The CLR served as a catalyst for enhancing coaches’ interpersonal and intrapersonal skills. Through engaging with the CLR, the coaches felt more aware of the FRLM (Bass and Riggio, 2006) and were able to connect the behaviours to their own specific experiences. Increased awareness of the leadership behaviours was an important outcome of the intervention, as having the ability to identify the leadership dimension that they—or another coach—are displaying may contribute to more mindful interpersonal interactions with athletes. Moreover, the CLR appeared to enhance coaches’ self-awareness of areas for improvement in their leadership behaviours by providing valuable opportunities for outsider feedback and reflective discussion. Building on McGuckin’s (2016) use of the CLAS for observation and video recall, the present study incorporated CLAS-GRS feedback directly into debrief conversations, encouraging deep reflection on coaching behaviours.

In addition, the study’s findings suggest that coaches were directly prompted by the CLR to recognize their need for greater reflection on interpersonal interactions, rather than technical/tactical factors. Coaches’ realization during the CLR suggests a prior lack of awareness of what specifically they were reflecting on. This is unsurprising, as Nash et al. (2022) found that coaches are often unaware of whether they engage in reflective practice at all and generally lack understanding of how to reflect. The current findings align with and extend this research, revealing that when coaches do reflect, it tends to focus largely on technical/tactical components rather than interpersonal interactions. As coaches take greater care and consideration in reflecting on their leadership behaviours, they will – hopefully – display more transformational behaviours. In doing so, coaches will be more effective (Côté and Gilbert, 2009) and ultimately contribute to more positive coach-athlete relationships. This is extremely important for athletes’ long-term development (Erickson and Côté, 2015; Fraser-Thomas et al., 2005; Jowett and Cockerill, 2003; Petitpas et al., 2008), improved athletic performance (Phillipe and Seiler, 2006), and satisfaction with sport experiences (Jowett and Nezlek, 2011); all of which are key factors in athletes’ continued sport participation (Côté et al., 2022).

The CLR played a significant role in empowering coaches by enhancing their confidence in their reflective practice, coaching philosophies, and current coaching behaviours. Evidently, the structured nature of the CLR was instrumental in effectively guiding coaches through reflective practice; offering a targeted reflective focus through the pre-brief/debrief questioning and the post-training reflection card, and allowing for individualized reflection through goal setting and CLAS-GRS observation graphs. The CLR, particularly the knowledge of the FRLM (Bass and Riggio, 2006) and the r-card, provides coaches with the capabilities to engage in interpersonal reflection on their own, ultimately leading to increased confidence in their reflective skills. As previously discussed, a key factor in one’s motivation and behaviour is their self-efficacy, which is determined by several aspects, including “mastery experience” (Bandura, 1982). Mastery experience occurs when one has successfully completed a task, which in turn increases belief in one’s ability to complete similar tasks in the future. Similarly, coaching efficacy (i.e., CE) refers to the extent that coaches feel they can influence their athletes’ learning and performance (Feltz et al., 1999). Providing coaches with an opportunity to reflect on their interpersonal behaviours through the CLR successfully may serve as a source for CE and future reflective practice.

In addition, the findings show that coaches gained confidence in their current leadership behaviours through the CLR, specifically due to the pre-brief session. Outlining the leadership dimensions allowed coaches to create an informal ‘mental checklist’ in which participants reflected on their engagement with specific leadership behaviours. Doing so resulted in self-assurance that they were “on the right track,” meaning that coaches displayed behaviours consistent with their coaching values. Answering Lefebvre et al.’ (2023) call to utilize the CLAS-GRS to facilitate feedback discussions and stimulate self-reflection, the CLR provides the opportunity for feedback based on in-situ observation and reflection on the alignment of their displayed behaviours and their coaching philosophies. This notion may be especially beneficial for new coaches whose coaching philosophies are being defined. Accordingly, Nash et al. (2008) found that newer or inexperienced coaches are less likely to be aware of or consider their coaching values or philosophies than more experienced coaches.

Further, the current findings demonstrate coaches’ drive to implement aspects of the CLR and their learning into their regular coaching practices. For instance, coaches reported feeling motivated to reflect more frequently, specifically by employing the post-training reflection card. This particular outcome of the CLR is significant given the known positive impact of reflection on both coach development and favourable athlete outcomes (Cropley et al., 2015; Nelson and Cushion, 2006; Whitehead et al., 2016). This finding is also crucial, as it suggests the CLR fulfills its intended purpose of prompting reflection and developing coaches’ intrapersonal skills.

### Limitations and implications

To provide a holistic view of this project, it is imperative to acknowledge the potential limitations associated with this study. Firstly, the coach participants were recruited from competitive volleyball clubs located in southern Ontario, Canada. Consequently, the generalizability of the study’s findings may be limited and future scholarship may investigate the applicability of these findings to other contexts, sports (e.g., basketball, soccer, rugby) and participant demographics. At the same time, the inclusion of coaches from multiple competitive clubs provided perspectives across varied team environments, offering a meaningful foundation for understanding the intervention within the context of youth competitive sport. However, the small sample size (*N* = 12) of participants may have contributed to a deceiving sense of data saturation. Additionally, given that the interviewer was also the CLR facilitator, participants may have experienced social desirability bias, potentially leading them to provide more positive feedback in the interview than they otherwise would have. Although, several factors help mitigate this concern. Participants provided both positive and negative feedback throughout the interviews and offered numerous constructive comments, suggesting a willingness to engage critically and reflect candidly on their experiences. However, it is recommended that future research may employ distinct facilitators and interviewers to mitigate potential biases.

## Conclusion

This study aimed to address the call for qualitative research examining the CLAS-GRS’ ability to foster feedback discussions that support coaches’ interpersonal development (Lefebvre et al., 2023), while also responding to recommendations to collaborate with youth sport-invested partners (i.e., coaches) when developing and evaluating CDP opportunities (Da Silva et al., 2020; Evans et al., 2015). The findings from this study provide meaningful insights into coaches’ interpersonal and intrapersonal development, the factors that facilitate and hinder reflective practice, and the potential real-world application of the CLR intervention. These findings contribute to future research on structured reflective tools, long-term coach and athlete outcomes, and the development of youth sport coaching policies/certification and educational programming. Practically, coaches’ reflections on the broader implications of the CLR for sport suggest that it may positively impact both individual coaches and sport organizations as a whole (e.g., providing a structured reflection process to enhance mentorship discussions or current coach evaluations). Based on coaches’ feedback on the CLR outcomes, the present findings indicate that the CLR will serve as a valuable resource for youth sport coaches and help improve coaching practices and the overall sport context.

## Data Availability

The original contributions presented in the study are included in the article/supplementary material, further inquiries can be directed to the corresponding author.
